# Developing Biomarkers of Mild Traumatic Brain Injury: Promise and Progress of CNS-Derived Exosomes

**DOI:** 10.3389/fneur.2021.698206

**Published:** 2022-02-10

**Authors:** Melonie N. Vaughn, Charisse N. Winston, Natalie Levin, Robert A. Rissman, Victoria B. Risbrough

**Affiliations:** ^1^Department of Neurosciences, University of California, San Diego, San Diego, CA, United States; ^2^Veterans Affairs San Diego Health System, University of California, San Diego, San Diego, CA, United States; ^3^Department of Psychiatry, University of California, San Diego, San Diego, CA, United States; ^4^VA Center of Excellence for Stress and Mental Health, La Jolla, CA, United States

**Keywords:** exosome, mild traumatic brain injury, biomarkers, cytokines, miRNA

## Abstract

Mild traumatic brain injuries (mTBI) are common injuries across civilian and military populations. Although most individuals recover after mTBI, some individuals continue to show long-term symptoms as well as increased risk for neurodegenerative and neuropsychiatric disorders. Currently, diagnosing TBI severity relies primarily on self-report and subjective symptoms, with limited tools for diagnosis or prognosis. Brain-derived exosomes, a form of extracellular vesicle, may offer a solution for interpreting injury states by aiding in diagnosis as well as outcome prediction with relatively low patient burden. Exosomes, which are released into circulation, contain both protein and RNA cargo that can be isolated and quantified, providing a molecular window into molecular status of the exosome source. Here we examined the current literature studying the utility of exosomes, in particular neuronal- and astrocyte-derived exosomes, to identify protein and miRNA biomarkers of injury severity, trajectory, and functional outcome. Current evidence supports the potential for these emerging new tools to capture an accessible molecular window into the brain as it responds to a traumatic injury, however a number of limitations must be addressed in future studies. Most current studies are relatively small and cross sectional; prospective, longitudinal studies across injury severity, and populations are needed to track exosome cargo changes after injury. Standardized exosome isolation as well as advancement in identifying/isolating exosomes from CNS-specific tissue sources will improve mechanistic understanding of cargo changes as well as reliability of findings. Exosomes are also just beginning to be used in model systems to understand functional effects of TBI-associated cargo such as toxicity. Finally linking exosome cargo changes to objective markers of neuronal pathology and cognitive changes will be critical in validating these tools to provide insights into injury and recovery states after TBI.

## Introduction

Traumatic brain injury (TBI) is the leading cause of death and disability in the United States and in the developing world (1). TBI results from a bump, blow, jolt, or any other external force to the head that disrupts normal brain function (2, 3). In the United States, nearly 2 million people sustain a TBI annually, contributing to one-third of all injury-related deaths (4). TBI is a significant cause of mortality in children and young adults, and the incidence in older individuals has increased with the average life span. There are three basic levels of TBI injury: mild, moderate, and severe. Mild TBIs (mTBI), e.g., concussions, are the most frequent type to result in post-TBI survival (5). Historically, the term concussion refers to a low-velocity injury that results in the rapid onset of short-lived impairment of neurological function that resolves spontaneously (5). However, in some cases, symptoms and signs may evolve over a number of hours to days (6). It's important to note that the temporary loss of brain function manifests in a graded set of clinical symptoms that may or may not involve loss of consciousness (LOC) (7). mTBIs may result in neuropathological changes, but the acute clinical symptoms largely reflect a functional disturbance rather than a structural injury and, as such, rarely is an abnormality observed using standard structural neuroimaging. Because of this, often times mTBIs go undiagnosed since traditional computed tomography scans (CT) and magnetic resonance imaging (MRI) may fail to capture milder injuries which do not qualify as contusions or hematomas (7, 8). Overall, the majority of mTBI patients do eventually recover from their injuries; however up to 20–50% may experience long-term symptoms (9–12). TBI also results in increased risk for development of other brain disorders, including neuropsychiatric and neurodegenerative disorders (13–16). TBI is also associated with significantly increased prevalence of developing mood and anxiety disorders, including suicidal ideation, major depression and post-traumatic stress disorder (PTSD) (13, 16).

The incidence of the growth of TBI injuries in American has disproportionally affected the Veteran population. Over 300,000 Veterans have sustained TBIs during combat and training (8, 17) and due to the mild symptomology, mTBI injuries are grossly underreported. In Veterans, TBI is particularly challenging to treat because clinicians must treat TBI symptoms years after the initial injury. There is limited access to medical information about the initial insult, and acute symptoms and are typically what is used to inform severity and potential treatment strategies. Identifying accessible and clinically feasible biomarkers for TBI is essential to appropriate diagnosis of TBI-associated dysfunction and to develop appropriate intervention strategies.

Similar to neurodegenerative diseases, there are several biomarkers for TBI derived from neuroimaging modalities (8, 18) and cerebral spinal fluid (CSF) sampling (19). CSF is the chief biological source for identifying CNS-specific biomarkers of disease. However, CSF sampling is expensive, invasive, and not readily available in some medical settings, reducing its clinical feasibility. While neuroimaging modalities are less invasive, they are similarly costly to implement and have limited access for some communities. Because of the resources and expertise required, timing between injury, and CSF/imaging collection can impeded proper identification of mTBI patients who are underinsured or living in underserved, rural areas, making it harder to track chronic symptoms (20). These limitations have fueled research into alternative biomarkers derived from accessible biofluids. Blood-based biomarkers of TBI are relatively easy and inexpensive to collect with minimal equipment required, non-invasive, and can be relatively high-throughput. A number of promising blood-based TBI biomarkers have emerged in addition to CSF markers, including tau, neurofilament light chain (NFL), glial fibrillary acidic protein (GFAP), ubiquitin carboxyl-terminal hydrolase isoenzyme L1 (UCHL-1), neuron-specific enolase, myelin basic protein, and calcium-binding protein as well as potential multi-variate proteomic panels (19, 21, 22). Free circulating microRNAs (miRNA) have also been identified as potential markers of head injury (23, 24). However, blood levels of free circulating proteins and mRNA are easily degraded and thus can fall below threshold detection levels for identification. The tissue source is also unknown, which limits interpretation of potential mechanistic contributions. In an effort to circumvent these challenges, recent studies have suggested that circulating exosomes enriched for CNS-specific tissue sources may be a tool to identify accessible biomarkers for TBI (25, 26).

**Figure 1:**
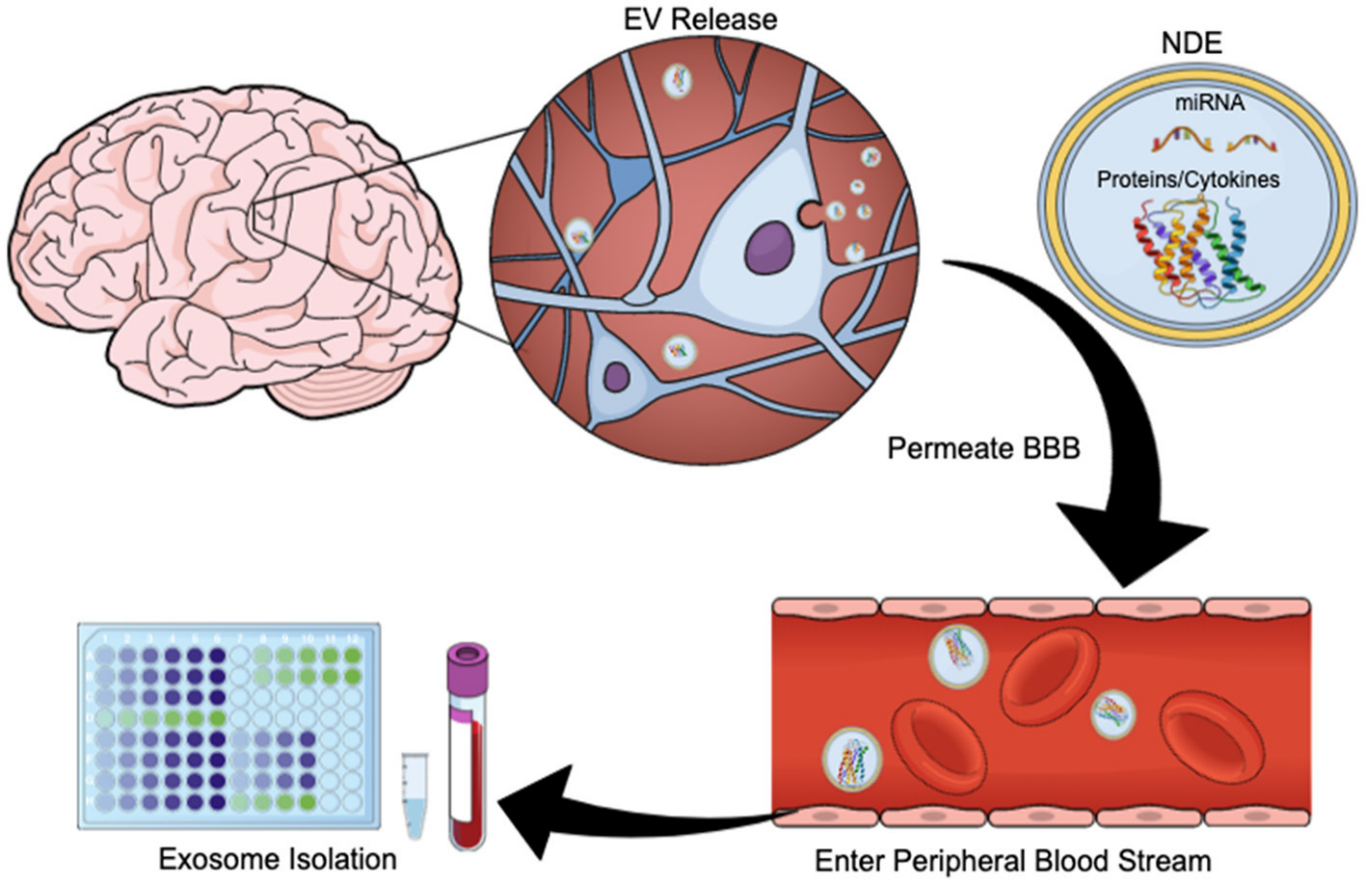
Exosome Release Schematic. EVs are released by neurons and glia. Their cargo often contains protein, cytokines, and/or miRNA. These exosomes can cross the blood brain barrier and enter the peripheral bloodstream, allowing for quantification and study. This figure was created using the Mind the Graph online platform (www.mindthegraph.com).

Exosomes are membrane-bound, extracellular vesicles (EVs) that are released from cells upon fusion of multivesicular bodies with the plasma membrane, and play a direct role in intercellular communication by carrying proteins and RNA (including messenger RNA, microRNAs, small nuclear RNA, non-coding RNA, and small cytoplasmic RNA) between cells (27, 28). Exosomes have diameters ranging from 40 to 100 nm and contain membrane-bound proteins associated with cellular origin (29). CNS-derived exosomes cross the blood-brain barrier (BBB) and are readily extracted from peripheral blood (Figure 1). Using immunoprecipitation techniques exosome preparations can be enriched for unique membrane-bound proteins associated with neuronal and astrocyte sources (neuronal- and astrocyte derived exosomes, NDEs, ADEs, respectively) (25, 30, 31). Cargo from exosomes enriched for NDEs are being tested for diagnostic and prognostic utility for neurodegenerative disorders such as Alzheimer's disease (AD), Frontotemporal dementia, Parkinson's disease, Down Syndrome and Huntington's disease (31–35). NDE cargo is examined for proteins associated with neurodegeneration or synapse loss. Protein cargo from both NDEs and ADEs can accurately differentiate patients with dementia from age-matched controls (36) and are predictive of MCI conversion to AD (30, 37). This is a rapidly evolving field and these tools are now being used to identify biomarkers of acute and chronic sequelae of mTBI (26, 38–40).

The clinical utility of blood-based exosomes is predicated on the fact that cargo proteins are protected from degradation via the exosome's phospholipid bilayer as compared to free circulating proteins and RNA (41, 42). Given that EVs can be enriched for CNS-derivation and are readily detected in blood, they hold substantial promise to providing an accessible substrate to identify biomarkers and mechanics of neuropathology. However, as the field currently stands, exosome isolation methods are numerous, which can limit reproducibility (43, 44). Classic isolation methods include ultracentrifugation methods and column chromatography (45). These methods tend to generate a relatively homogenous population of EVs, however the source of these exosomes are not known. In order to assess exosome cargo that are reflective of a specific cellular origin, typically commercially available kits are used to first precipitate exosomes from blood, followed by targeted enrichment methods which include, but not limited to, magnetic bead immunocapture against a neural marker, such as L1CAM and/or the astrocytic marker, glutamine aspartate transporter. Subsequently, exosome immunocapture preparations are tagged with an exosome surface marker and sorted using fluorescent activated cell sorting (FACS) (25, 37).

Here we will review currently identified protein and miRNA cargo from extracellular vesicles associated with TBI and functional outcome across military and civilian populations. We will discuss how the diverse contents of exosome cargo are particularly influenced by their cellular source (total exosome vs. cellular sub-populations) and how that impacts their biomarker potential for TBI. Table 1 summarizes all protein biomarkers identified thus far, and classifies them according to severity, temporal significance, and significantly associated cognitive or mental disorders.

**Table 1:** Human plasma protein markers.

**Marker**	**Mi**	**Mo**	**Sev**	**rTBI (>2)**	**Acute**	**Sub-Acute**	**Chronic**	**Pop**	**Exosome**	**Related**	**References**
					**(H/Day)**	**(Days/Wks)**	**(Mths/Yrs)**	**type**	**Pop**	**symptoms**	
p-Tau	↑	↑	↑	↑		↑	↑	M, C	NDE, Total	PCS, PTSD, CI	(25^*^, 39, 40, 46–48)
Total Tau	↑	↑	↑	↑	↑	Slightly lower, still Elevated	↑	M, C	NDE, Total	PCS, PTSD, CI	(25^*^, 38–40, 47, 49, 50)
Aβ-42	↑	↑	↑	↑		↑	↑	M, C	NDE, ADE	CI	(25, 38, 40, 48, 51, 50^*^)
PrPc	↑	↑	↑			↑	↑	M, C	NDE	CI	(40, 48)
synaptogyrin-3	↑	↑	↑			↑	↑	M, C	NDE	CI	(40, 48)
NRGN	↓			↓			↓	M	NDE, ADE		(25)
IL6	↑	↑	↑			↑	↑	M, C	NDE	CI	(40, 46, 48, 50, 52^*^)
IL10	↑	↑		↑			↑	M	NDE	PTSD	(38, 50^*^, 46^*^, 52^*^)
TNFα	↑						↑	M	Total	CI	(38^*^, 50^*^, 46, 52^*^)
CD59, CR1	↓	↓				↓	↓	M, C	ADE		(53)
Factor D, Bb	↑	↑				↑	↑	M, C	ADE		(53)
C3b, C5b-9	↑	↑				↑	↑	M, C	ADE		(53)
GFAP		↑	↑		↑	Normal	↑	M, C	Total, NDE	CI	(46, 49, 54)
Aquaporin- 4	↑		↑		↑	Slightly lower, still elevated	Normal	M, C	NDE, Total		(40, 48, 54)
NFL	↑	↑	↑	↑	↑	↑	↑	M, C	Total, NDE	PCS, PTSD, MDD, CI	(25^*^, 46, 49, 50, 52)
UCHL1	↑	↑	↑		↑	↑	Normal	C	NDE, Total		(40, 46^*^)
Annexin-VII	↑			Normal		↑	Normal	C	NDE		(25, 40, 48^*^)
Spectrin Fragments	↑					↑	Normal	C	NDE		(40)
Claudin-5	↑					↑	Normal	C	NDE		(40, 48^*^)
NKCC-1	↑					↑	Normal	C	NDE		(40)

In this table, ^*^ indicates the study cited did not completely validate the findings of others in said category. M, military; C, civilian; Mi, mild TBI; Mo, moderate TBI; Sev, Severe TBI; rTBI, repetitive TBI; Pop, population; NKCC, Na-K-Cl cotransporter. Other abbreviations are used as previously described. ↑, increased; ↓, decreased after injury.

This review was conducted using the following search terms in pubmed, “((exosome) OR extracellular vesicles OR exosomal) AND (biomarker) AND ((tbi) OR (traumatic brain injury)).” This yielded 68 results across all years (earliest found dated back to 2014). Of these results, several were not original research. Review articles and perspective articles were filtered out. Original Studies were then omitted based on the following criteria: they did not engage with blood based-biomarkers or blood-derived exosomes, or they did not present original findings for the disease of interest (must relate to TBI). Individual case studies with <3 TBI subjects were also removed. Our review included papers which were left after applying the aforementioned criteria. This process was repeated on Google Scholar. All of the search strategies were performed from the initiation to April 2021, and the literature was published in English. The references of selected articles were further searched by hand to obtain additional citations.

## Neurodegenerative Proteins

### Total Tau and P-Tau

The most consistently assessed protein biomarkers for TBI are total tau and phosphorylated tau. Tau is a protein associated with neurodegenerative diseases such as AD and related dementias (55). In non-pathological conditions, tau maintains cytoarchitecture of neurons, and axons and dendritic spines of neurons via microtubule stabilization (56). However, in tauopathies like AD, clusters and tangles of hyper-phosphorylated and misfolded tau aggregate at synaptic sites and disrupt neuronal function and integrity (57). Similar tau tangles are observed in brains of patients with chronic traumatic encephalopathy (CTE) (58). CTE is induced after repeated mTBIs, resulting in axonal and white matter fiber degeneration (59). Tau is released into the CSF and blood following axonal injury (60), with tau and phosphorylated forms of tau (p-tau) detected in plasma after severe TBI as well as in some cases chronically (61–63). Interpretation of circulating tau is limited however because it is not only from CNS sources but to a lesser extent expressed in peripheral tissues such as muscle, kidney and lung (64). CNS-derived exosomes may provide a complementary approach to assess CNS-specific sources of tau.

Initial studies examining exosome cargo for TBI associated changes in tau used total exosome preparations (51). Total exosome preparations were obtained from plasma samples from former National Football League players who exhibited symptoms in line with a likely CTE diagnosis (CTE diagnosis is confirmed post-mortem). Participants underwent prior evaluation to determine if they met the diagnostic criteria for CTE before participating (65). Repetitive mild TBI, was associated with increased total tau in exosome cargo in comparison to age-matched controls in non-impact sports (*N* = 78 TBI, *N* = 16 controls). Exosome tau levels had 82% sensitivity, 100% specificity for predicting TBI, with 100% positive predictive value but limited negative predictive value (53%). Exosome tau was negatively correlated with neuropsychological performance suggesting relationship with functional outcomes. Plasma levels of tau were not found to be significantly elevated in the TBI group suggesting examining plasma levels without including exosome analysis could miss or mischaracterize abnormal protein levels.

Findings of increased total tau or p-tau in total exosomes were in line with known severe tauopathies associated with post-mortem CTE. However, several studies have since identified total tau protein and/or phosphorylated tau associations with mild or isolated TBI events using CNS-derived exosome enrichment strategies. Elevated levels of exosome total tau has been observed in mild, moderate, severe, and repetitive TBI patients ranging from days to years after the initial injury (38–40, 47, 49, 51). The consistency in findings of increased tau levels across TBI severity suggests tau is a relatively sensitive marker despite the heterogeneous nature of TBI presentations. The time course of increased tau levels appears to follow both an acute and a chronic trajectory. Moderate to severe TBI patients exhibit immediate elevation in total exosome tau that decrease by ~5-fold within 5 days after injury, supporting tau as a marker of acute injury (49). However, another study examined cargo enriched for NDEs, finding that that chronic (i.e., 3–12 months after injury) mTBI patients had higher NDE total tau and p-tau levels than those with acute injuries (40). Finally, a number of studies have shown increased p-tau and or total tau in NDEs and total exosomes of patients many years after TBI (39, 40, 48). Overall, these results indicate a potential temporal aspect should be considered when measuring accumulations of tau in neuronal derived exosomes. A study from our lab found no change in ADE or NDE p-tau r tau levels within ~3–6 months of injury in service members reporting mild TBIs during a combat deployment, although other markers of axonal injury and synaptic loss were present (25). Additionally, a comparative study in Veterans with chronic mTBI did not find group differences for total exosome levels of tau, although past work from this group has shown higher levels of tau are associated with repetitive mTBIs (39, 50). Taken together, the data suggest that tau increases may follow both an acute and chronic course [e.g., see (66)]. Initial tau increases in exosomes are in line with tau-release after acute neuronal damage. The secondary rise in p-tau and tau however likely reflects secondary pathology associated with chronic neurodegenerative processes such as a rise in amyloid beta (see below).

### Aβ42

Amyloid beta 42 (Aβ42) is also a common marker associated with TBI. Aβ42 is a product of amyloid precursor protein, which is normally located in the synaptic membranes of neurons (67). Amyloid precursor protein undergoes cleaving which can lead to the accumulation of Aβ isoforms, such as Aβ42, and the formation of plaques in the brain. These Aβ plaques are believed to contribute to neurotoxicity and the progression of AD. TBI can result in Aβ accumulation in the soma and axon of neurons, which may contribute to prolonged neuronal injury (68). Additionally, severe head injuries increase the risk for AD, suggesting a potential overlap in etiology (69).

Similar to tau, multiple studies have shown that Aβ42 is elevated in isolated exosomes amongst every clinical classification of TBI (mild, moderate, severe, and repetitive) spanning from days to many years after initial injury (25, 38, 40, 48). Increased Aβ42 has been detected in both neuronal- and astrocyte-enriched exosome samples from individuals with TBI (25, 38, 40, 48). There were two studies that failed to detect group differences in Aβ42 levels: one investigated NDEs, and the other study examined total exosomes in Veterans (46, 50). Guedes et al. had relatively fewer controls and examined total exosome not an enriched sample for CNS-specific exosomes which may explain lack of detection (*N* = 28 control, 71 TBI, 45 TBI + PTSD). Peltz et al. however did enrich for NDEs and had a relatively large sample size compared to other studies with positive results, suggesting neither power or exosome sample would be at issue (*N* = 60 control, 95 TBI (46) vs. *N* = 42 control, *N* = 47 TBI (48). However, the mean age of participants amongst cohorts in Peltz et al. was 76–82 years. With one exception, studies reporting significant elevations of Aβ42 included participants with mean ages ranging from 19 to 30 years at time of collection (25, 38, 40). This difference in age could account for their failure to detect elevated Aβ42 levels in Veterans with chronic mTBI, as NDE levels of Aβ42 could fluctuate with age, with Aβ42 rising with age (70).

These findings demonstrate the potential for exosome total tau, p-tau, and Aβ42 levels to be used as biological markers for TBI however it will depend on tissue source of exosomes and potentially age range. Both civilian and military populations were considered in studies examining these markers, suggesting these changes are not tied to military-specific injuries (e.g., blast injuries). In order to further validate these findings, studies with larger sample sizes would be most beneficial, as would longitudinal studies to understand if early increases in these markers predict later neuropathology and/or chronic symptom course, as well as understand when over the TBI trajectory these markers are most detectable. Finally, it should be noted that although group differences are detected, however the ability of these markers to provide diagnostic specificity is relatively untested at this point due to relatively small sample sizes and retrospective nature of most study designs.

### Cytokines

In addition to assessing the biomarker potential of NDEs in TBI, recent studies are emerging to assess the biomarker potential of cargo proteins extracted from exosomes derived from astrocytes (ADEs) and other glial sources (30, 36, 37). Neuroinflammation is a well-characterized pathological hallmark of TBI (71). Initially, CNS and peripherally derived inflammatory cells respond to TBI to provide some amount of neuroprotection (72). However, inflammatory mediators have been implicated in both delayed CNS damage, impacting neurological recovery (73). Astrocytes mediate these inflammatory responses by release of inflammatory cytokines into the extracellular environment (74) and thus ADE cargo may aid identification of CNS inflammation.

Cytokines, including interleukins, are released by immune cells as well as other immune modulators to coordinate immune response to tissue injury or infection. Astrocytes express multiple cytokine receptors as well as produce and release cytokines in response to cellular damage (75). Their overexpression or dysregulation contributes to chronic and acute neuroinflammation, and in the case of TBI, secondary damages after initial injuries (76–78). The inflammatory cytokines interleukin-6 (IL-6) and Tumor necrosis factor-alpha (TNFα) in addition to the anti-inflammatory cytokine interleukin-10 (IL-10) have been measured in exosomes of TBI patients. The elevation of these cytokines in NDEs and ADEs suggests abnormal regulation of neuroinflammation the potential presence of secondary injuries as a result of a TBI.

IL-6 is elevated in NDEs and total exosomes of patients with mild, moderate, and severe forms of TBI (40, 46, 48). IL-6 increases are detected both at sub-acute and chronic time points, suggesting it may be indicative of long-lasting inflammation in response to the initial injury. Data on acute NDE levels of IL-6 (hours up to 1 day after injury) were not available, so it is unclear how long IL-6 takes to appear in the exosome cargo. Total exosome levels of pro-inflammatory TNFα were also observed to be significantly elevated in military Veterans suffering from chronic TBI (46). However, three studies failed to replicated these findings when examining total exosome levels in similar Veteran populations suffering from chronic mild or moderate cases (38, 50, 52). Conversely, while Gill et al. (38) found an elevation of NDE levels of anti-inflammatory cytokine IL-10, (50, 52) and Peltz et al. (46) did not.

This discrepancy could be due to variations in the methodologies used by each study. While Guedes et al. examined total exosome levels of cytokines, Peltz et al. and Gill et al. examined only neuron-derived exosomes. These differences amongst tissue source of exosomes may explain why the findings varied across groups. Additionally, even though all studies examined Veterans with chronic TBI, Guedes et al. and Gill et al. studied participants with median ages in their 30s while Peltz et al. examined patient groups with a median age of late 70s/early 80s. Time since injury and overall age could account for some of the differences observed since several exosome markers have been shown to be temporally sensitive. Finally, method of exosome isolation (ultracentrifugation vs. Exoquick) also appears to affect cytokine profiles from exosomes, suggesting that even initial exosome isolations methods can bias exosome fraction types and or affect protein cargo quantification steps (79). Other validation steps may also be required to ensure that cytokine cargo being measured is from CNS-derived exosome cargo, as markers used to target NDEs are also expressed by immune cells. More work needs to be done to standardize and confirm cytokine profiles in TBI populations, however this work may yield important progress in delineating accessible markers of CNS-specific inflammation after brain injury. Identifying CNS inflammation also has potential prescriptive value in the future, in identifying subpopulations for specific interventions targeted at immune regulation.

## Injury Markers: GFAP, NFL, Aquaporin 4, UCHL-1

### NFL

Multiple studies investigated the expression levels of neuronal and glial injury markers within the exosome profiles of individuals with TBI. One of the most studied markers is the structural scaffolding protein neurofilament light chain (NFL). NFL is abundantly expressed in long myelinated axons, and abnormal levels of NFL are released into the extracellular space upon axonal injury cause by neurodegeneration and other forms of trauma (80). Increases of NFL levels in cerebrospinal fluid (CSF) have been associated with dementias, AD, multiple sclerosis, stroke, Parkinson's disease, Huntington's disease and TBI (80, 81). In total exosomes isolated from plasma, NFL is increased in mild, moderate, and severe TBI patients at several time points after injury in comparison to controls (46, 49, 50, 52). On the acute and sub-acute level, amounts of NFL found in exosomes increased steadily in the 5 days following injury (49). Number of TBIs sustained and years since last TBI was also positively correlated with increases in total exosome and plasma NFL levels in Veterans (52). In contrast, one study in active duty personnel ~6–12 mo after TBI failed to detect higher NFL in NDEs, however it was significantly smaller and did not have as many participants endorsing repeated head injuries (25). These results suggest exosome NFL is detectable both acutely and remotely after TBI. It is not clear however if particular exosome populations (total, vs. NDE or ADE) vs. plasma measures may provide more sensitive and consistent NFL measurements of TBI exposure and related axonal injury/neurodegeneration associated with a chronic course.

### GFAP

Glial fibrillary acidic protein (GFAP) is a cytoarchitectural protein in astrocytes, and its detection in blood serum or CSF is commonly used as indicator of glial injury (82). For these reasons, studies investigating spinal cord injuries, TBI, stroke, AD, and Parkinson's disease commonly look at GFAP as a biomarker of neurodegeneration and acute astrocytic injury. Significant elevations in exosome GFAP levels of TBI patients are reported (46, 54). Levels of GFAP were found to be highest immediately following moderate to severe TBI (49, 54). However, GFAP levels declined as early as 48 h after injury and continued falling up to 5 days later (49, 54). Although these findings were from preparations of total exosomes or extracellular vesicles, GFAP is only expressed in the cytoplasm of astrocytes, thus it can be assumed these signals came from ADEs (83). Although GFAP levels fall immediately after injury, long lasting elevations in GFAP are associated with long-term cognitive impairment in Veterans with TBI (46). It is not clear, however, if GFAP is associated with mild or repeated TBI.

### UCH-L1

Ubiquitin carboxy-terminal hydrolase L1 (UCH-L1) is found in the cytoplasm of neurons and has been shown to be necessary for the maintenance of axonal and neuronal health (84). Alterations in blood serum levels of UCH-L1 has predictive value for the recovery outcome (as measured by Glasgow Outcome Scale) of patients suffering from TBIs (85). UCH-LI is also higher in NDEs within 7 days of mild TBI (40), but not chronically. In a temporal profile study with moderate to severe TBI patients, levels of UCH-L1 in total exosomes were shown to decline within 24 h of injury (49). Median levels of total exosome levels of UCH-L1 were 8-fold higher on the day of injury in comparison to those observed on day 5, a significant decrease. It should be noted that this study did not include a control comparison, limiting interpretation of whether these shifts reflect significant changes from variance in healthy controls. Thus, there is limited evidence that UCH-LI is increased in exosomes immediately after injury, but temporal changes, relationship with severity and number of injuries is unknown.

### AQP4

Aquaporin-4 (AQP4) is a water channel protein found in the end-feet of astrocytes and may contribute to neuroinflammatory processes and edema production (86). NDE and total exosome levels of AQP4 are elevated in both mild and severe TBI (40, 54). Goetzl et al. (40) found that NDE levels of AQP4 were significantly elevated in both chronic and acute mild TBI patients compared to controls. While this elevation stayed significant across all timepoints measured, it did appear to slightly decrease between 1 week and 3–12 months after injury. When investigating EVs of multiple types, Nekludov et al. (54) found significantly elevated levels of AQP4 in severe TBI patients during their first 3 days post-injury. In a sample of chronic TBI patients, AQP4 differences in NDEs were not detected (48), suggesting AQP4 may only be detectable relatively acutely after injury.

## Potential Predictors of Functional Outcome

Although biomarkers have the potential to identify neurological insults, their clinical utility would be significantly strengthened if they can be used to predict functional outcomes such as cognitive decline or mental health symptoms. TBI significantly increases the risk for PTSD, depression, and other mental illnesses (13, 14, 16). Many patients living with chronic TBIs also report enduring symptoms of post-concussive syndrome (PCS) or cognitive impairment (CI). Although relatively small in sample size, several studies examined the relationship between behavioral function and exosome cargo.

Cognitive impairment was the most frequently examined functional outcome associated with exosome protein cargo. Nine different proteins have been identified as correlating significantly with severity of cognitive impairment. Cellular prion protein (PrPc), p-tau, synaptogyrin-3, and Aβ42 were elevated in NDEs of TBI patients with CI (*N* = 26 with previous TBI, *N* = 19 without), but not in healthy controls (*N* = 42) nor TBI patients without CI symptoms (*N* = 21) (48). In this case, CI was determined by administered cognitive tests which measured general cognitive abilities, verbal memory, and mental processing speed (Mini Mental State Examination, Auditory Verbal Learning Task, and Wechsler Adult 24 Intelligence Scale-Revised Digit Symbol Task, respectively). These findings are in line with post-mortem studies of excess neuronal p-tau and tau associations with cognitive decline (87). IL-6 was identified by the same study as being correlated with only cognitively-impaired chronic-TBI patients, and not cognitively-impaired, no-TBI controls. A separate study reported IL6, NFL, TNF-alpha, and GFAP were elevated in NDEs of TBI patients with CI (*N* = 35) compared to TBI patients without CI (*N* = 30) (46). However, only p-tau was significantly elevated in CI TBI patients in comparison to CI controls (*N* = 30). This study determined CI using the previously described cognitive tests, and/or current dementia diagnoses. Most studies are limited to examining exosome cargo relationships with cognitive impairment at the group level, due to relatively small sample size. One study has shown total exosome total tau levels correlated significantly with cognitive functioning as measured by short-term memory (Neuropsychological Assessment Battery) and psychomotor speed (Wechsler Adult Intelligence Scale-Revised) in retired professional football players (*N* = 78) (51). Perhaps not surprisingly, these data suggest that both neurodegenerative and inflammatory protein cargo are not only associated with TBI over controls, but more be most detectable in TBI populations that exhibit CI. There are no data yet, however, on how exosome cargo might predict future cognitive decline after TBI.

In addition to CI, there is some limited evidence that NDE tau and NFL markers are also associated with PCS symptoms (39, 52), which include self-reported sleep disturbances, memory deficits, attentional deficits, mood changes, headaches, irritability, and fatigue. Several studies have also identified exosome cargo links with neuropsychiatric symptoms. Elevated NDE and total exosome levels of p-tau and total tau in patients with repetitive and mild TBIs is associated with increased PTSD symptoms (38, 39). Elevated NDE and total exosome levels of IL-10 and NFL are also associated with PTSD or depression symptoms in chronic mTBI (38, 50, 52). Thus, far it appears that these markers are relatively non-specific in relation to functional outcomes in cognitive and psychiatric domains, however caution is warranted at this early stage given the relatively small sample sizes in these studies. Larger studies will be needed to differentiate NDE exosome cargo associated with TBI-specific cognitive impairment over associations with aging and other comorbidities (e.g., neuropsychiatric disorders, other mechanisms of neurodegeneration).

Future longitudinal analysis of how exosome cargo changes over time in conjunction with the presentation and resolution of these symptoms will aid inferring causality. Finally, while these markers alone may have relatively low predictive value for diagnostic uses, future combination of exosome cargo markers with other moderators of neurodegenerative risk, (i.e., polygenic risk scores for neurodegenerative disorders or Apolipoprotein E (APOE) genotype) may be useful in conjunction with exosome cargo proteins in developing predictive markers of functional outcome such as long-term cognitive decline after TBI.

## miRNA Cargo Associated With TBI

MicroRNAs (miRNA) are non-coding RNAs, which bind to messenger RNAs to regulate gene expression, often via post-transcriptional slicing. miRNA can be packaged into exosomes and transported to various parts of the body where they interact with and are taken up by recipient cells (88). Exosome miRNAs can then functionally alter the recipient cell by altering gene expression levels within the cell. In some instances, miRNAs can bind to toll-like receptors in immune cells and activate an inflammatory response (89). Due to their ability to manipulate gene expression and induce systemic inflammation, miRNAs have recently gained interest as an exosome cargo with the potential to alter or induce pathogenic states. Table 2 summarizes the reviewed studies which found significant changes in miRNA associated with TBI. An exhaustive list of miRNA of interest is included in Supplementary Tables 1, 2. Some of the most promising candidates are described below, however most have been identified in preclinical models and have not yet been detected in human exosomes associated with TBI.

**Table 2:** miRNAs of interest across species.

**Study**	**Pop**.	***N***	**Trauma**	**Over-expressed MiRNAs**	**Under-expressed miRNAs**	**Timepoints**	**Exosome Source**	**NDE/ADE/Total**
Devoto et al. (90)	Human Military	*N* = 73 1–2 TBI, *N* = 35 control	Blast	miR-3190-3p, 18a-5p, 372-3p, **376b-3p**, **382-3p**, **375**	miR-139-5p*, **150-5p**	~11 yrs	Plasma	Total
Harrison et al. (91)	Mice, M 7–9 wks	*N* = 12, 3 pooled samples per group	CC	miR-7a-5p, 7b-5p, 15a-5p, 21a-5p, 130a-3p*, **142a-5p**, **146a-5p**, 181b-1-3p, 203-3p*, **219a.2-3p**	miR-30e-3p*, 128-2-5p*, 130-b-5p*, 139-3p*, 221-3p, 335-3p*, 361-3p*	7 days	Brain extract	Total
Huang et al. (92)	Mice, M 10–12 wks	*N* = 6/group	CCI, Rep TBI	miR-124-3p*, 124-3, 124-2, 128-3p*, 125a-5p, 138-5p, 376b-3p, 335, **382-5p**, **434-3p**	None	3, 7, 14, 21, 28 days	Brain extract	ADE
Ko et al. (93)	Human civilian	*N* = 16 TBI, *N* = 20 control	mild TBI	miR-185-5p, 206	miR-203a-3p*, 203b-5p	<24 hrs	Plasma	Total
Ko et al. (93)	Mice, M 12–14 wks	N = 56 CCI, 60 Blast, 36 control	Blast, CCI	miRNA-9-5p*, 219a.2-3p, 488-3p	miR-22-5p, **150-5p**, 351-3p, 669c-5p, 6236	1 h, 1, 4, 14 days	Plasma	Total
Gayen et al. (94)	Humans, *in vitro*	N/A	IL-6	miRNA-**29a**, 30d*, 130b*, 139-5p*, 141, 143, 145*, 146a, 194, 203*, 375	None	24 h	Human Astrocyte Supernatant	ADE
Ge et al. (95)	Mice, M 12 wks	*N* = 3 per group	CCI, Rep TBI	miRNA-124-3p* (3 & 14d), 6096-5p	miR-124-3p* (42d), 744-3p, 764-3p, 767, 7046-3p, 7660-3p, 7665-5p, 7674-5p	3, 14, 42 days	Brain extract	ADE
Wang et al. (96)	Rats, M 12–16 wks	*N* = 3 per group	Weight drop	miRNA-9a-3p*, 29b-3p, 124-3p*, 142-3p, 181c-3p, 195-3p, 328-5p, 361-3p*, 434-3p, 451-5p, 532-5p	miRNA-9a-5p, 28-3p, 92a-3p, 96-5p, 145-3p*, 221-5p, 222-3p	24 h	Plasma	Total
Guedes (50)	Human Military	*N* = 28 control *N* = 71 mTBI/-PTSD *N* = 45 mTBI/+PTSD	mTBI	**miR-3190-3p, 615-5p**, 1185-1-3p, 3196, 509-3-5p, **204-5p, 372-3p, 1277-3p**	**miR-139-5p**	~9 yrs	Plasma	Total

*In this table, bolded items represent miRNA identified in multiple studies. Items with an asterisk represent those found to be inversely altered across studies (i.e., over-expressed in one, under-expressed in another). M, Male; CCI, controlled cortical impact. Other abbreviations are as previously defined*.

### miRNA-124-3p

miR-124-3p is the most frequently identified exosome miRNA associated with TBI to date. miR-124-3p is significantly downregulated in Alzheimer's disease and in gliomas, suggesting an involvement in neurodegeneration and CNS disease (97, 98). Levels of astrocyte-derived exosome miR-124-3p are elevated in acute and subacute mouse models (3 and 14 days after injury) of controlled cortical impact (CCI) induced repetitive TBI (95). However, levels of miR-124-3p fell after day 14, becoming significantly under-expressed by 42 days after injury. In a preclinical model of TBI. miR-124-3p was elevated in mice from 3 to 21 days after repetitive CCI injury (92). This finding suggests temporal dependence of expression pattern, which may potentially contribute to differential presentations of TBI symptoms. In rats, miR-124-3p isolated from total exosomes are elevated 24 h after a weight drop injury (96).

To asses function, two studies transfected miR-124-3p mimics into cultured mouse microglia cells and used the resulting exosomes to treat injured cell lines (92, 95). Following repetitive scratch-injuries, treatment with the exosome miR-124-3p lead to increased branch numbers and neurite length restoration, and a decrease in p-tau and Aβ proteins (92, 95). These results suggest miR-124-3p may play a role in preventing neurodegeneration during the acute stages of injury recovery. miR-124-3p can inhibit Aβ mutations by targeting a transcription factor known as Rela (95), and also suppresses mTOR signaling via PDE4B inhibition, resulting in a decrease of neuroinflammation (92). This finding might explain why this miRNA is so frequently elevated immediately after injury, however this miRNA has not yet been identified in clinical studies of TBI, thus it is not clear if these findings translate to clinical outcomes.

### miRNA-146

miR-146 is significantly elevated in multiple TBI and exosome studies and has been previously linked to inflammatory diseases and signaling pathways. Levels of brain-derived exosome miR-146 are elevated in rhesus macaques suffering from simian immunodeficiency virus encephalitis (99). Additionally, miRNA-146 reduces inflammatory gene expression in dendritic cells and endotoxin-induced inflammation in mice (100). Total exosome levels of miRNA-146a-5p are significantly elevated 7 days after CCI induced TBI in mice (91). In humans, *in vitro* experiments demonstrate that astrocytes subjected to IL-6 stress injuries significantly overexpress astrocyte-derived exosome miRNA-146a within 24 h of treatment (94). These results suggest that miRNA-146 could regulate neuroinflammation following TBI. It is unclear whether levels of miRNA-146 are chronically elevated after injury or only for an acute time period. Increases in exosome miR-146 levels might have the potential to improve clinical outcomes, since they may aid in inhibiting inflammation in the brain. However, additional studies measuring effects in human patients are needed to gauge if these results are translatable.

### miRNA-30d

miR-30d increases the expression of pro-inflammatory cytokines and is a marker of severe inflammation (101). Increased levels of miR-30d in brain-derived exosomes could potentially indicate elevated levels of neural inflammation after TBI. Human astrocytes expressed increased levels of miR-30d 24 h after injury via IL-6 treatment (94). This finding supports previous results which show that blood serum levels of miR-30d are upregulated in mild, moderate, and severe TBI patients within 48 h after injury (102). Taken together, miR-30d may be acutely upregulated in human astrocytes following TBI, leading to activation or mediation of neuroinflammatory signaling cascades via the regulation of cytokines, further contributing to injury severity (94). A related miRNA, miR-30e-3p, was found to be under-expressed 7 days after CCI injury in mice (91). Although these miRNAs are in the same miRNA-30 family, it could be they play separate roles in neuroinflammation or injury response. Additionally, this study was carried out in rodents on a sub-acute timescale, thus it is not known if increased expression is limited to acute timepoints. This family has not yet been investigated clinically.

## Discussion/Future Studies

### Confounders and Population Considerations

While the current summary of findings from both protein and miRNA focused studies are promising, there are many considerations for improving interpretability of exosome-based research. There are a number of potential confounds that the field must address that are common problems for most biomarker studies. First, medications may alter exosome cargo, or ability of CNS-derived exosomes to travel into circulation. For example anti-inflammatory medications that affect immune function may alter cytokine levels in biofluids, biasing detection, and interpretation of cytokine and other immune markers (e.g., TNFα, IL-1β). Lifestyle choices will also potentially effect exosome cargo just as it does circulating protein and RNA profiles. For example cannabis, tobacco or alcohol, especially chronic use, will alter the circulating levels of cytokines (103, 104). Although it is not known if these lifestyle choices can affect CNS-specific cargo, it seems relatively likely given their effects on inflammation and cell metabolism [see (105)] and transcriptional markers. Most studies reviewed here do not account for a history of tobacco use. It is also not known how vascular abnormalities, whether genetically or environmentally pre-disposed, could alter exosome transport, suggesting future research would benefit from examining whether effects hold true when accounting for participants' use of antihypertensives. Similarly, understanding genetic factors that may predispose to certain exosome cargo could prove beneficial. For example, carriers of the APOE4 allele, which modulates plaque buildup and alters tau and Aβ42 levels in the brain may be pre-disposed to have higher levels of neurodegenerative exosome cargo (106). Adding genetic differences into consideration would make future data collected more powerful, as it would control for neurodegenerative protein cargo variations otherwise thought to be induced by head trauma. Of the papers reviewed here, none conducted genetic screenings for APOE4 genotypes or other genetic modifiers (i.e., polygenic risk scores for neurodegenerative diseases like AD). Finally, across current studies there is a large age range for the participants examined. As humans age, baseline levels of circulating tau, Aβ, and other markers of neurodegeneration will increase (107). Therefore, a marker for TBI which is useful in one population may prove to be unaltered in another.

Another element for supporting future clinical utility is that while most studies were conducted using a sample which was representative of their surrounding populations, they were not always representative of the ethnic and racial makeup of the United States at large. This lack of representation may affect generalizability of the results considering different racial and ethnic groups face different environmental pressures which may alter their ability to seek timely medical care following head injury (20) or alter the state of their vascular health (108), ultimately affecting their exosome cargo profile. Additionally, male participants are over-represented in most of the studies reviewed, especially those drawing from military populations. This is likely due to the sizable sex differences amongst soldiers in the combat fields. For example, less than two percent of women make up the infantry force of the US Army (109). However, as the integration of women into infantry becomes more commonplace this difference should reflect in sample populations. Every effort should be made in the field to study participants which represent every facet of American life.

### Injury Time Course Considerations

The studies reviewed above clearly support that most exosome cargo biomarkers, as with plasma and CSF markers, are dependent upon inter-injury interval (66). Table 1 illustrates the time course of exosome cargo protein levels, indicating the potential post injury trajectory of these markers. In most cases, the trajectory of these markers must also be inferred by different cross-sectional studies using different inter-injury intervals. Future research is needed to expand and improve resolution of this time course of exosome cargo biomarkers after injury if these markers are to be useful in clinical settings. Longitudinal assessment of blood-based markers is relatively feasible compared to CSF and imaging and will be critical for improving our understanding of the trajectory of each marker as well as allow for causal or predictive inferences for exosome-identified signaling pathways for chronic symptoms.

### Injury Type Considerations

While the studies reviewed did specify and describe the severity of injury in their subject populations, they failed to examine group differences between blast and non-blast injuries. This information is especially relevant when discussing exosome biomarkers obtained from military personnel. Due to the nature of combat military service, service members are at an increased risk of being exposed to explosive blast injuries compared to their civilian counterparts. Blast overpressure can cause head trauma through several proposed mechanisms: rotational/translational acceleration of the brain, reverberation of compression waves, and vascular surges (110). Although treatment for TBI does not differ by injury type, the mechanisms of injury and subsequent biomarkers induced by blast overpressure are likely distinct, in particular in terms of level of exposure of the brain to peripheral immune responses/infection through blood brain barrier disruption or penetrative injuries (111, 112). In regards to outcome measures, a systematic review found that long term recovery and symptoms of blast vs. non-blast groups were similar (113). However, small sample sizes, variable assessment measures, and inconsistent findings hinder firm conclusions. More information regarding injury type should be reported in future comparative studies in order to assess potential differences in clinical outcomes and circulating blood biomarkers of specific injury types.

### Methodological Considerations

Exosome cargo has strong potential for identifying biomarkers for diagnosis and prognosis of TBI, however a number of technical challenges will need to be overcome. Enriching for CNS-specific populations is a rapidly moving field, with different approaches each having different limitations and benefits (114). While conventional ultracentrifugation (UC)-based methods have been widely used to isolate exosomes, other techniques including size-exclusion chromatography (SEC), immunoaffinity capture, microfluidics, and poly-ethylene glycol (PEG)-based precipitation methods have emerged as suitable alternatives (115). Comparative studies suggest that methodological variations amongst the different techniques can drastically alter the type of exosomes and cargo that can be detected. Recently, the use of commercially available, PEG-based precipitant kits has facilitated higher extraction efficiency of exosomes as compared to UC or SEC. However, UC and SEC samples appear to have less protein contamination as compared to PEG-based samples. The chemical impurities from the precipitant may interfere with downstream applications and bias the interpretation of these findings. Secondary isolation methods to target exosomes from a specific biological sources (i.e., cell culture, brain, blood, CSF, urine, milk, etc.) can also bias findings as well as potentially affect the stability of the exosome cargo (114); which will affect reproducibility across studies. Currently, there is no standardized protocol that is suitable for every study and that is widely accepted. An in-depth investigation into the methodological issues associated with exosome isolation and tissue origins is warranted for the TBI field, to examine which methodologies yield the most robust and reliable signal. These comparative analyses are required to identify the best isolation method that is most suitable for a specific biological source and that maximizes the diagnostic and prognostic abilities of exosome cargo for TBI-relevant applications.

To date, most of the work has assessed the cargo composition and diagnostic potential of exosomes derived from neuronal exosomes. While a limited number of studies have expanded into exosomes derived from astrocytes, cell-specific markers for microglial and oligodendrocytes exosomes are still being developed. As technology improves for isolating specific exosome populations from other extracellular vesicle populations [for a recent review, see (44)], we will expect to see more refined and reliable candidate biomarkers emerge. It will be essential to understand how the diversity of exosome parental origins impact their cargo composition in order to develop clinically-viable markers of TBI pathology and progression.

## Author Contributions

VR, RR, CW, NL, and MV contributed to the conception of the review, coordinated writing efforts, and edited the final article version. CW, RR, and NL wrote the introduction. MV and VR wrote the subsequent sections: Neurodegenerative Proteins, cytokines, injury markers, predictors of functional outcome, and miRNA. MV, VR, RR, and CW wrote the discussion section. MV constructed the figure, and all tables. All authors contributed to revisions of the manuscript, approved the submitted version, and agree to be accountable for all aspects of the work.

## Funding

This study was supported by grants AG057459, AG057469, AG051848, AG018440, and ADRC P50 AG005131 from the National Institute on Aging, and VA Merit Awards BX003040 and BX004312 to RR and VA Merit Award BX004312, Department of Defense W81XWH1810761, P50 MH096889-06 to VR, VA Merit Award RX002484 to CW, University of California Eugene Cota-Robles Fellowship Award to MV, and the VA Center of Excellence for Stress and Mental Health funding to VR.

## Conflict of Interest

The authors declare that the research was conducted in the absence of any commercial or financial relationships that could be construed as a potential conflict of interest.

## Publisher's Note

All claims expressed in this article are solely those of the authors and do not necessarily represent those of their affiliated organizations, or those of the publisher, the editors and the reviewers. Any product that may be evaluated in this article, or claim that may be made by its manufacturer, is not guaranteed or endorsed by the publisher.
