# Supplementary material for: Developing Biomarkers of Mild Traumatic Brain Injury: Promise and Progress of CNS-Derived Exosomes
Source: Front Neurol. 2022 Feb 10;12:698206. doi: 10.3389/fneur.2021.698206 (PMC8866179; doi:10.3389/fneur.2021.698206)
Supplement: Supplementary file 1 [file Table_1.pdf]

miRNA Family	Subtype	Population	Trauma	Timeframe	Exosome Source	NDE/ADE/Total	Ref
7	a-5p	Mice, M, 7-9wks	CCI	7 Days	Brain Extract	Total	Harrison, 2016
7	b-5p	Mice, M, 7-9wks	CCI	7 Days	Brain Extract	Total	Harrison, 2016
let-7	d	Human, In Vitro	IL6 Stimulation	24 hrs	Astrocyte Supernatant	ADE	Gayen, 2020
let-7	e	Human, Chronic, Vet	Blast	~11 years	Plasma	Total	Devoto, 2021
**9***	5p	Mice, M, 12-14wks	Blast, CCI	1 hr, 1, 4, 14 day	Plasma	Total	Ko, 2019
**9**	a-3p	Rats, M, 12-16wks	Weight Drop	24 hrs	Plasma	Total	Wang, 2020
15	a-5p	Mice, M, 7-9wks	CCI	7 Days	Brain Extract	Total	Harrison, 2016
15	b-5p	Mice, M, 7-9wks	CCI	7 Days	Brain Extract	Total	Harrison, 2016
18a	5p	Human, Chronic, Vet	Blast	~11 years	Plasma	Total	Devoto, 2021
21	a-5p	Mice, M, 7-9wks	CCI	7 Days	Brain Extract	Total	Harrison, 2016
27	a	Human, In Vitro	IL6 Stimulation	24 hrs	Astrocyte Supernatant	ADE	Gayen, 2020
**29**	a	Human, In Vitro	IL6 Stimulation	24 hrs	Astrocyte Supernatant	ADE	Gayen, 2020
**29**	b-3p	Rats, M, 12-16wks	Weight Drop	24 hrs	Plasma	Total	Wang, 2020
30*	d	Human, In Vitro	IL6 Stimulation	24 hrs	Astrocyte Supernatant	ADE	Gayen, 2020
**31**		Human, In Vitro	IL6 Stimulation	24 hrs	Astrocyte Supernatant	ADE	Gayen, 2020
**31**	5p	Human, Chronic, Vet	Blast	~11 years	Plasma	Total	Devoto, 2021
92	b-3p	Mice, M, 7-9 wks	CCI	7 Days	Brain Extract	Total	Harrison, 2016
93		Human, In Vitro	IL6 Stimulation	24 hrs	Astrocyte Supernatant	ADE	Gayen, 2020
100	5p	Mice, M, 10-12wks	CCI, Rep TBI	3, 7, 14, 21, 28 Days	Brain Extract	ADE	Huang, 2017
106	b-5p	Rats, M, 12-16wks	Weight Drop	24 hrs	Plasma	Total	Wang, 2020
107		Human, Chronic, Vet	Blast	~11 years	Plasma	Total	Devoto, 2021
**124***	3p	Mice, M, 10-12wks	CCI, Rep TBI	3, 7, 14, 21, 28 Days	Brain Extract	ADE	Huang, 2017
124	1	Mice, M, 10-12wks	CCI, Rep TBI	3, 7, 14, 21, 28 Days	Brain Extract	ADE	Huang, 2017
124	3	Mice, M, 10-12wks	CCI, Rep TBI	3, 7, 14, 21, 28 Days	Brain Extract	ADE	Huang, 2017
124	2	Mice, M, 10-12wks	CCI, Rep TBI	3, 7, 14, 21, 28 Days	Brain Extract	ADE	Huang, 2017
**124**	3p	Mice, M, 12 wks	CCI, Rep & mTBI	3, 14 Days	Brain Extract	ADE	Ge, 2020
**124**	3p	Rats, M, 12-16wks	Weight Drop	24 hrs	Plasma	Total	Wang, 2020
125	a-5p	Mice, M, 10-12wks	CCI, Rep TBI	3, 7, 14, 21, 28 Days	Brain Extract	ADE	Huang, 2017
126		Human, In Vitro	IL6 Stimulation	24 hrs	Astrocyte Supernatant	ADE	Gayen, 2020
127	3p	Mice, M, 10-12wks	CCI, Rep TBI	3, 7, 14, 21, 28 Days	Brain Extract	ADE	Huang, 2017
128*	3p	Mice, M, 10-12wks	CCI, Rep TBI	3, 7, 14, 21, 28 Days	Brain Extract	ADE	Huang, 2017

miRNA Family	Subtype	Population	Trauma	Timeframe	Exosome Source	NDE/ADE/Total	Ref
**130***	a-3p	Mice, M, 7-9 wks	CCI	7 Days	Brain Extract	Total	Harrison, 2016
**130**	**b**	Human, In Vitro	IL6 Stimulation	24 hrs	Astrocyte Supernatant	ADE	Gayen, 2020
**133**	**a-3p**	Mice, M, 7-9 wks	CCI	7 Days	Brain Extract	Total	Harrison, 2016
**133**	**b-3p**	Mice, M, 7-9 wks	CCI	7 Days	Brain Extract	Total	Harrison, 2016
138	5p	Mice, M, 10-12wks	CCI, Rep TBI	3, 7, 14, 21, 28 Days	Brain Extract	ADE	Huang, 2017
**139***	**5p**	Human, In Vitro	IL6 Stimulation	24 hrs	Astrocyte Supernatant	ADE	Gayen, 2020
141		Human, In Vitro	IL6 Stimulation	24 hrs	Astrocyte Supernatant	ADE	Gayen, 2020
**142**	**a-5p**	Mice, M, 7-9 wks	CCI	7 Days	Brain Extract	Total	Harrison, 2016
**142**	**3p**	Rats, M, 12-16wks	Weight Drop	24 hrs	Plasma	Total	Wang, 2020
143		Human, In Vitro	IL6 Stimulation	24 hrs	Astrocyte Supernatant	ADE	Gayen, 2020
**145***		Human, In Vitro	IL6 Stimulation	24 hrs	Astrocyte Supernatant	ADE	Gayen, 2020
**146**	**a-5p**	Mice, M, 7-9 wks	CCI	7 Days	Brain Extract	Total	Harrison, 2016
**146**	**a**	Human, In Vitro	IL6 Stimulation	24 hrs	Astrocyte Supernatant	ADE	Gayen, 2020
151	3p	Mice, M, 7-9 wks	CCI	7 Days	Brain Extract	Total	Harrison, 2016
152		Human, In Vitro	IL6 Stimulation	24 hrs	Astrocyte Supernatant	ADE	Gayen, 2020
**181**	**b-1-3p**	Mice, M, 7-9 wks	CCI	7 Days	Brain Extract	Total	Harrison, 2016
**181**	**c-3p**	Rats, M, 12-16wks	Weight Drop	24 hrs	Plasma	Total	Wang, 2020
185	5p	Human, Civilian	Mild TBI	1 day	Plasma	Total	Ko, 2019
192		Human, In Vitro	IL6 Stimulation	24 hrs	Astrocyte Supernatant	ADE	Gayen, 2020
193	a-3p	Mice, M, 7-9 wks	CCI	7 Days	Brain Extract	Total	Harrison, 2016
194		Human, In Vitro	IL6 Stimulation	24 hrs	Astrocyte Supernatant	ADE	Gayen, 2020
**195**		Human, In Vitro	IL6 Stimulation	24 hrs	Astrocyte Supernatant	ADE	Gayen, 2020
**195**	**3p**	Rats, M, 12-16wks	Weight Drop	24 hrs	Plasma	Total	Wang, 2020
197		Human, In Vitro	IL6 Stimulation	24 hrs	Astrocyte Supernatant	ADE	Gayen, 2020
**200**	**b**	Human, In Vitro	IL6 Stimulation	24 hrs	Astrocyte Supernatant	ADE	Gayen, 2020
**200**	**c**	Human, In Vitro	IL6 Stimulation	24 hrs	Astrocyte Supernatant	ADE	Gayen, 2020
**203***	**3p**	Mice, M, 7-9 wks	CCI	7 Days	Brain Extract	Total	Harrison, 2016
**203**		Human, In Vitro	IL6 Stimulation	24 hrs	Astrocyte Supernatant	ADE	Gayen, 2020
**204**	**5p**	**Human, Chronic Vet**	**Blast**	**~11 years**	**Plasma**	**Total**	**Devoto, 2021**
**204**	**5p**	**Human, Chronic Vet**	**mTBI**	**~9 years**	**Plasma**	**Total**	**Guedes, 2021**

miRNA Family	Subtype	Population	Trauma	Timeframe	Exosome Source	NDE/ADE/Total	Ref
206		Human, Civilian	Mild TBI	1 day	Plasma	Total	Ko, 2019
215		Human, In Vitro	IL6 Stimulation	24 hrs	Astrocyte Supernatant	ADE	Gayen, 2020
218	3p	Mice, M, 10-12wks	CCI, Rep TBI	3, 7, 14, 21, 28 Days	Brain Extract	ADE	Huang, 2017
**219**	**a-5p**	Mice, M, 7-9 wks	CCI	7 Days	Brain Extract	Total	Harrison, 2016
**219**	**a-2-3p**	Mice, M, 7-9 wks	CCI	7 Days	Brain Extract	Total	Harrison, 2016
**219**	**a.2-3p**	Mice, M, 12-14 wk	Blast, CCI	1 hr, 1, 4, 14 day	Plasma	Total	Ko, 2019
300	5p	Mice, M, 7-9 wks	CCI	7 Days	Brain Extract	Total	Harrison, 2016
328	a-5p	Rats, M, 12-16wks	Weight Drop	24 hrs	Plasma	Total	Wang, 2020
335*	5p	Mice, M, 10-12wks	CCI, Rep TBI	3, 7, 14, 21, 28 Days	Brain Extract	ADE	Huang, 2017
335		Mice, M, 10-12wks	CCI, Rep TBI	3, 7, 14, 21, 28 Days	Brain Extract	ADE	Huang, 2017
338	3p	Mice, M, 7-9 wks	CCI	7 Days	Brain Extract	Total	Harrison, 2016
346		Human, Chronic, Vet	Blast	~11 years	Plasma	Total	Devoto, 2021
350	3p	Mice, M, 7-9 wks	CCI	7 Days	Brain Extract	Total	Harrison, 2016
361*	3p	Rats, M, 12-16wks	Weight Drop	24 hrs	Plasma	Total	Wang, 2020
**372**	**3p**	**Human, Chronic Vet**	**Blast**	**~11 years**	**Plasma**	**Total**	**Devoto, 2021**
**372**	**3p**	**Human, Chronic Vet**	**mTBI**	**~9 years**	**Plasma**	**Total**	**Guedes, 2021**
374	5p	Rats, M, 12-16wks	Weight Drop	24 hrs	Plasma	Total	Wang, 2020
**375**		Human, In Vitro	IL6 Stimulation	24 hrs	Astrocyte Supernatant	ADE	Gayen, 2020
**375**		Human, Chronic, Vet	Blast	~11 years	Plasma	Total	Devoto, 2021
**376**	b-3p	Mice, M, 10-12wks	CCI, Rep TBI	3, 7, 14, 21, 28 Days	Brain Extract	ADE	Huang, 2017
**376**	b-3p	Human, Chronic, Vet	Blast	~11 years	Plasma	Total	Devoto, 2021
**382**	**3p**	Human, Chronic, Vet	Blast	~11 years	Plasma	Total	Devoto, 2021
**382**	**5p**	Mice, M, 10-12wks	CCI, Rep TBI	3, 7, 14, 21, 28 Days	Brain Extract	ADE	Huang, 2017
**434**	3p	Mice, M, 10-12wks	CCI, Rep TBI	3, 7, 14, 21, 28 Days	Brain Extract	ADE	Huang, 2017
**434**	3p	Rats, M, 12-16wks	Weight Drop	24 hrs	Plasma	Total	Wang, 2020
450b	3p	Human, Chronic, Vet	Blast	~11 years	Plasma	Total	Devoto, 2021
488	3p	Mice, M, 12-14 wk	Blast, CCI	1 hr, 1, 4, 14 day	Plasma	Total	Ko, 2019
494		Human, In Vitro	IL6 Stimulation	24 hrs	Astrocyte Supernatant	ADE	Gayen, 2020
495	3p	Mice, M, 10-12wks	CCI, Rep TBI	3, 7, 14, 21, 28 Days	Brain Extract	ADE	Huang, 2017
509	3-5p	Human, Chronic Vet	mTBI	~9 years	Plasma	Total	Guedes, 2021

miRNA Family	Subtype	Population	Trauma	Timeframe	Exosome Source	NDE/ADE/Total	Ref
520	d-3p	Human, In Vitro	IL6 Stimulation	24 hrs	Astrocyte Supernatant	ADE	Gayen, 2020
532	5p	Rats, M, 12-16wks	Weight Drop	24 hrs	Plasma	Total	Wang, 2020
539		Human, In Vitro	IL6 Stimulation	24 hrs	Astrocyte Supernatant	ADE	Gayen, 2020
542	3p	Mice, M, 7-9 wks	CCI	7 Days	Brain Extract	Total	Harrison, 2016
567		Human, Chronic, Vet	Blast	~11 years	Plasma	Total	Devoto, 2021
**615**	**5p**	**Human, Chronic Vet**	**Blast**	**~11 years**	**Plasma**	**Total**	**Devoto, 2021**
**615**	**5p**	**Human, Chronic Vet**	**mTBI**	**~9 years**	**Plasma**	**Total**	**Guedes, 2021**
885	5p	Human, In Vitro	IL6 Stimulation	24 hrs	Astrocyte Supernatant	ADE	Gayen, 2020
1183		Human, Chronic, Vet	Blast	~11 years	Plasma	Total	Devoto, 2021
1233	3p	Human, Chronic, Vet	Blast	~11 years	Plasma	Total	Devoto, 2021
**1277**	**3p**	**Human, Chronic Vet**	**Blast**	**~11 years**	**Plasma**	**Total**	**Devoto, 2021**
**1277**	**3p**	**Human, Chronic Vet**	**mTBI**	**~9 years**	**Plasma**	**Total**	**Guedes, 2021**
1543	3p	Mice, M, 7-9 wks	CCI	7 Days	Brain Extract	Total	Harrison, 2016
1185	1-3p	Human, Chronic Vet	mTBI	~9 years	Plasma	Total	Guedes, 2021
2887	2-3p	Mice, M, 7-9 wks	CCI	7 Days	Brain Extract	Total	Harrison, 2016
3099	3p	Mice, M, 7-9 wks	CCI	7 Days	Brain Extract	Total	Harrison, 2016
**3190**	**3p**	**Human, Chronic Vet**	**Blast**	**~11 years**	**Plasma**	**Total**	**Devoto, 2021**
**3190**	**3p**	**Human, Chronic Vet**	**mTBI**	**~9 years**	**Plasma**	**Total**	**Guedes, 2021**
3196		Human, Chronic Vet	mTBI	~9 years	Plasma	Total	Guedes, 2021
3470	b-3p	Mice, M, 7-9 wks	CCI	7 Days	Brain Extract	Total	Harrison, 2016
3959	3p	Mice, M, 7-9 wks	CCI	7 Days	Brain Extract	Total	Harrison, 2016
5001	5p	Human, Chronic, Vet	Blast	~11 years	Plasma	Total	Devoto, 2021
5099	3p	Mice, M, 7-9 wks	CCI	7 Days	Brain Extract	Total	Harrison, 2016
6096	5p	Mice, M, 12 wks	CCI, Rep & mTBI	3, 14, 42 Days	Brain Extract	ADE	Ge, 2020
6395		Mice, M, 7-9 wks	CCI	7 Days	Brain Extract	Total	Harrison, 2016

Supplemental Table 1: Overexpressed miRNA across species. This table details miRNA which has been identified as undergoing a significant increase in expression levels after traumatic brain injury. M = male, Vet = Veteran, CCI = controlled cortical impact, Rep TBI = repetitive TBI. Items in bold are miRNA families identified in multiple studies. Items with * have been identified as being significantly under-expressed by a different study (**listed in Supplemental Table 2**).
