# Supplementary material for: Developing Biomarkers of Mild Traumatic Brain Injury: Promise and Progress of CNS-Derived Exosomes
Source: Front Neurol. 2022 Feb 10;12:698206. doi: 10.3389/fneur.2021.698206 (PMC8866179; doi:10.3389/fneur.2021.698206)
Supplement: Supplementary file 2 [file Table_2.pdf]

miRNA Family	Subtype	Pop	Trauma	Time Point(s)	Exosome Source	NDE/ADE Total	Source
**9***	**a-5p**	Rats, M, 12-16wks	Weight Drop	24hrs	Plasma	Total	Wang, 2020
22	5p	Mice, M, 12-14wks	Blast, CCI	1hr, 1, 4, 14 day	Plasma	Total	Ko, 2019
26	a-5p	Mice, M, 7-9wks	CCI	7 Days	Brain Extract	Total	Harrison, 2016
28	3p	Rats, M, 12-16wks	Weight Drop	24hrs	Plasma	Total	Wang, 2020
**30***	**e-3p**	Mice, M, 7-9wks	CCI	7 Days	Brain Extract	Total	Harrison, 2016
**30**	**a-3p**	Mice, M, 7-9wks	CCI	7 Days	Brain Extract	Total	Harrison, 2016
92	a-3p	Rats, M, 12-16wks	Weight Drop	24hrs	Plasma	Total	Wang, 2020
96	5p	Rats, M, 12-16wks	Weight Drop	24hrs	Plasma	Total	Wang, 2020
**124***	**3p**	Mice, M, 12wks	CCI, Rep	42 Days	Brain Extract	ADE	Ge, 2020
**128***	**2-5p**	Mice, M, 7-9wks	CCI	7 Days	Brain Extract	Total	Harrison, 2016
**128**	**1-5p**	Mice, M, 7-9wks	CCI	7 Days	Brain Extract	Total	Harrison, 2016
**128**	**3p**	Mice, M, 7-9wks	CCI	7 Days	Brain Extract	Total	Harrison, 2016
**130***	**b-5p**	Mice, M, 7-9wks	CCI	7 Days	Brain Extract	Total	Harrison, 2016
**139***	**3p**	Mice, M, 7-9wks	CCI	7 Days	Brain Extract	Total	Harrison, 2016
**139**	**5p**	Human, Chronic Vet	Blast	~11 years	Plasma	Total	Devoto, 2021
**139**	**5p**	Human, Chronic Vet	mTBI	~9 years	Plasma	Total	Guedes 2021
**145***	**3p**	Rats, M, 12-16wks	Weight Drop	24hrs	Plasma	Total	Wang, 2020
**150**	**5p**	Mice, M, 12-14wk	Blast, CCI	1hr, 1, 4, 14 day	Plasma	Total	Ko, 2019

**150**	**5p**	Human, Chronic, Vet	Blast	~11 years	Plasma	Total	Devoto, 2021
**203***	**b-5p**	Human, Civilian	Mild TBI	1 day	Plasma	Total	Ko, 2019
**203**	**a-3p**	Human, Civilian	Mild TBI	1 day	Plasma	Total	Ko, 2019
212	5p	Mice, M, 7-9wks	CCI	7 Days	Brain Extract	Total	Harrison, 2016
**221**	**3p**	Mice, M, 7-9wks	CCI	7 Days	Brain Extract	Total	Harrison, 2016
**221**	**5p**	Rats, M, 12-16wks	Weight Drop	24hrs	Plasma	Total	Wang, 2020
222	3p	Mice, M, 7-9wks	CCI	7 Days	Brain Extract	Total	Harrison, 2016
229	b-3p	Mice, M, 7-9wks	CCI	7 Days	Brain Extract	Total	Harrison, 2016
298	5p	Mice, M, 7-9wks	CCI	7 Days	Brain Extract	Total	Harrison, 2016
**335***	**3p**	Mice, M, 7-9wks	CCI	7 Days	Brain Extract	Total	Harrison, 2016
337	5p	Mice, M, 7-9wks	CCI	7 Days	Brain Extract	Total	Harrison, 2016
351	3p	Mice, M, 12-14wks	Blast, CCI	1hr, 1, 4, 14 day	Plasma	Total	Ko, 2019
**361***	**3p**	Mice, M, 7-9wks	CCI	7 Days	Brain Extract	Total	Harrison, 2016
370	5p	Mice, M, 7-9wks	CCI	7 Days	Brain Extract	Total	Harrison, 2016
451	5p	Rats, M, 12-16wks	Weight Drop	24hrs	Plasma	Total	Wang, 2020
669	c-5p	Mice, M, 12-14wk	Blast, CCI	1hr, 1, 4, 14 day	Plasma	Total	Ko, 2019
671	5p	Mice, M, 7-9wks	CCI	7 Days	Brain Extract	Total	Harrison, 2016
744	3p	Mice, M, 12wks	CCI, Rep	3, 14, 42 Days	Brain Extract	ADE	Ge, 2020
764	3p	Mice, M, 12wks	CCI, Rep	3, 14, 42 Days	Brain Extract	ADE	Ge, 2020
767		Mice, M, 12wks	CCI, Rep	3, 14, 42 Days	Brain Extract	ADE	Ge, 2020

770	5p	Mice, M, 7-9wks	CCI	7 Days	Brain Extract	Total	Harrison, 2016
874	3p	Mice, M, 7-9wks	CCI	7 Days	Brain Extract	Total	Harrison, 2016
940	5p	Mice, M, 7-9wks	CCI	7 Days	Brain Extract	Total	Harrison, 2016
1251		Mice, M, 7-9wks	CCI	7 Days	Brain Extract	Total	Harrison, 2016
1843	b-5p	Mice, M, 7-9wks	CCI	7 Days	Brain Extract	Total	Harrison, 2016
3473	e	Mice, M, 7-9wks	CCI	7 Days	Brain Extract	Total	Harrison, 2016
3547	3p	Mice, M, 7-9wks	CCI	7 Days	Brain Extract	Total	Harrison, 2016
4332	5p	Mice, M, 7-9wks	CCI	7 Days	Brain Extract	Total	Harrison, 2016
6215	l	Mice, M, 7-9wks	CCI	7 Days	Brain Extract	Total	Harrison, 2016
6236		Mice, M, 12-14wks	Blast, CCI	1hr, 1, 4, 14 day	Plasma	Total	Ko, 2019
6239	5p	Mice, M, 7-9wks	CCI	7 Days	Brain Extract	Total	Harrison, 2016
6240	5p	Mice, M, 7-9wks	CCI	7 Days	Brain Extract	Total	Harrison, 2016
6481	3p	Mice, M, 7-9wks	CCI	7 Days	Brain Extract	Total	Harrison, 2016
7641		Mice, M, 7-9wks	CCI	7 Days	Brain Extract	Total	Harrison, 2016
7641	2-5p	Mice, M, 7-9wks	CCI	7 Days	Brain Extract	Total	Harrison, 2016
7641	2-3p	Mice, M, 7-9wks	CCI	7 Days	Brain Extract	Total	Harrison, 2016
7660	3p	Mice, M, 12wks	CCI, Rep	3, 14, 42 Days	Brain Extract	ADE	Ge, 2020
7665	5p	Mice, M, 12wks	CCI, Rep	3, 14, 42 Days	Brain Extract	ADE	Ge, 2020
7674	5p	Mice, M, 12wks	CCI, Rep	3, 14, 42 Days	Brain Extract	ADE	Ge, 2020
7681	3p	Mice, M, 12wks	CCI, Rep	3, 14, 42 Days	Brain Extract	ADE	Ge, 2020
7977		Mice, M, 7-9wks	CCI	7 Days	Brain Extract	Total	Harrison, 2016

8962	5p	Mice, M, 7-9wks	CCI	7 Days	Brain Extract	Total	Harrison, 2016
9077	5p	Mice, M, 7-9wks	CCI	7 Days	Brain Extract	Total	Harrison, 2016
9342	5p	Mice, M, 7-9wks	CCI	7 Days	Brain Extract	Total	Harrison, 2016

Supplemental Table 2: Underexpressed miRNA across species. This table details miRNA which has been identified as undergoing a significant decrease in expression levels after traumatic brain injury. M = male, Vet = Veteran, CCI = controlled cortical impact, Rep TBI = repetitive TBI. Items in bold are miRNA families identified in multiple studies. Items with * have been identified as being significantly under-expressed by a different study (listed in **Supplemental Table 1**).
